# Capnovolumetry in combination with clinical history for the diagnosis of asthma and COPD

**DOI:** 10.1038/s41533-020-00190-z

**Published:** 2020-07-30

**Authors:** C. Kellerer, K. Klütsch, K. Husemann, S. Sorichter, R. A. Jörres, A. Schneider

**Affiliations:** 1 Technical University of Munich, School of Medicine, Institute of General Practice and Health Services Research, Munich, Germany; 2 Institute and Outpatient Clinic for Occupational, Social and Environmental Medicine, Ludwig-Maximilians-Universität München, Munich, Germany; 3 Medizinisches Versorgungszentrum MVZ Klinikum, Kempten, Germany; 4 RKK-Klinikum, St. Josefskrankenhaus, Freiburg, Germany

**Keywords:** Respiratory signs and symptoms, Asthma, Chronic obstructive pulmonary disease

## Abstract

Capnovolumetry performed during resting ventilation is an easily applicable diagnostic tool sensitive to airway obstruction. In the present analysis, we investigated in which way capnovolumetric parameters can be combined with basic anamnestic information to support the diagnosis of asthma and COPD. Among 1400 patients of a previous diagnostic study, we selected 1057 patients with a diagnosis of asthma (*n* = 433), COPD (*n* = 260), or without respiratory disease (*n* = 364). Besides performing capnovolumetry, patients answered questions on symptoms and smoking status. Logistic regression analysis, single decision trees (CHAID), and ensembles of trees (random forest) were used to identify diagnostic patterns of asthma and COPD. In the random forest approach, area/volume of phase 3, dyspnea upon strong exertion, s3/s2, and current smoking were identified as relevant parameters for COPD vs control. For asthma vs control, they were wheezing, volume of phase 2, current smoking, and dyspnea at strong exertion. For COPD vs asthma, s3/s2 was the primary criterion, followed by current smoking and smoking history. These parameters were also identified as relevant in single decision trees. Regarding the diagnosis of asthma vs control, COPD vs control, and COPD vs asthma, the area under the curve was 0.623, 0.875, and 0.880, respectively, in the random forest approach. Our results indicate that for the diagnosis of asthma and COPD capnovolumetry can be combined with basic anamnestic information in a simple, intuitive, and efficient manner. As capnovolumetry requires less cooperation from the patient than spirometry, this approach might be helpful for clinical practice.

## Introduction

Capnovolumetry has been proposed as a method to obtain information on the functional state of patients with obstructive airway diseases^1–4^. Capnovolumetric measurements are not time consuming and relatively easy to perform since the patient only needs to perform quiet tidal breathing over about 10 breathing cycles. Thus it is a technical method with low demands regarding cooperation. A further advantage of capnovolumetry is that the technique is already integrated in some of the commercially available spirometers without additional costs. The CO_2_ concentration in the exhaled air can be estimated from ultrasound signals by software algorithms without the need for an additional CO_2_ sensor, and ultrasound spirometers do not need to be calibrated. In contrast to spirometry, there is no need to give detailed instructions for forced breathing maneuvers by the technical personnel. Therefore, it is of special interest in conditions where spirometry is unreliable due to insufficient cooperation by the patients^5^ or lack of experience of the personnel in guiding the maneuvers or even concerns regarding the accuracy of spirometers^6^. Previous studies have shown a moderate diagnostic accuracy of this method regarding airway obstruction^1^ and an acceptability of spirograms for clinical use in only about 60% of patients in a primary care setting^6^. However, the establishment of a clinical diagnosis also includes clinical history, signs, and symptoms, which can be covered by a set of standard questions. Capnovolumetry might be combined with this information in the diagnostic set-up of obstructive airway diseases to increase diagnostic discrimination, similar to biomarkers that are effective in the diagnosis of specific conditions, including asthma^7–9^. There are several methods to achieve this integration, one of them being the construction of decision trees following objective statistical criteria. Such trees are well suited for clinical purposes^10^ and have been used, e.g., for the recognition of malignant lesions in magnetic resonance mammography^11^ or the identification of patients at risk from heart failure^12^. Decision trees also seem promising in the diagnosis of asthma and chronic obstructive pulmonary disease (COPD)^13^. Single decision trees computed by established techniques illustrate the structure of the decision process; however, as such trees are prone to overfitting, systematically constructed sets of independent trees (e.g., random forest) can be used to check the validity of the results.

Based on these arguments, we examined in which way questions regarding clinical history, signs, and symptoms could be best combined with capnovolumetric parameters in the diagnostic work-up of asthma and COPD. For this purpose, we used a large data set from a diagnostic study^1^ in which we had addressed the ability of capnovolumetry for the detection of airway obstruction without reference to the underlying diagnosis.

## Results

### Baseline characteristics

A total of 1400 consecutive patients underwent capnovolumetry. Patients who turned out to have had bronchial provocation challenges or bronchodilator testing prior to capnovolumetry due to organizational reasons were excluded (*n* = 45). Moreover, patients who did not undergo bodyplethysmographic and spirometric measurements (*n* = 61) were excluded. Five patients were excluded due to low quality of their bodyplethysmographic measurement data and two patients based on invalid capnovolumetric measurements. For the present analysis, patients were selected who had a diagnosis of COPD or asthma (or potentially overlap) or did not show any respiratory disease (control subjects). Two hundred and thirty patients with the diagnosis of other respiratory diseases (such as restrictive disorders, pneumonia or other infections, pleural diseases, lung tumor, bronchiectasis) were excluded (see Supplementary Fig. S1). Therefore, 1057 patients were analyzed, 567 (53.6%) were female and mean age was 56 years. Four hundred and thirty-three (41.0%) patients had a diagnosis of asthma, 260 (24.6%) COPD, and 364 (34.4%) were control patients (Table 1). Based on the lung function criteria used in our previous analysis^1^, 347 patients had airway obstruction. Of these, 108 (31%) had asthma, 223 (64%) had COPD, and 16 (5%) belonged to the control group. Thus 37 patients received a COPD diagnosis by pneumologists without actual airway obstruction.

**Table 1:** Baseline characteristics.

Parameter	Diagnostic groups			Comparison between groups
	Control	Asthma	COPD	*p* value
Gender (M/F)	172/192	155/278	163/97	<0.001
Age (years)	55 (41; 67)	53 (38; 63)	66 (57; 75)	<0.001
BMI (kg/m^2^)	26.9 (23.9; 30.9)	26.9 (24.1; 31.1)	26.6 (22.8; 30.5)	0.239
FEV_1_*Z*-score	−0.13 (−0.88; 0.53)	−0.82 (−1.53; −0.07)	−2.56 (−3.35; −1.78)	<0.001
FEV_1_/FVC *Z*-score	0.10 (−0.51; 0.76)	−0.68 (−1.36; 0.10)	−2.58 (−3.56; −1.67)	<0.001
FVC *Z*-score	−0.21 (−0.92; 0.45)	−0.38 (−1.14; 0.35)	−1.32 (−2.19; −0.60)	<0.001
Log_10_(s3)	−0.72 (−0.96; −0.52)	−0.72 (−0.92; −0.52)	−0.57 (−0.72; −0.39)	<0.001
Log_10_(s3/s2)	−1.00 (−1.10; −0.85)	−1.00 (−1.10; −0.89)	−0.80 (−0.92; −0.64)	<0.001
Area/volume phase 3 (g/mol)	0.05 (0.04; 0.07)	0.06 (0.04; 0.07)	0.08 (0.06; 0.10)	<0.001
Volume phase 2 (ml)	110.0 (92.0; 130.0)	102.0 (86.3; 121.8)	111.0 (95.0; 131.0)	<0.001
Current smoking	19.3% positive	11.6% positive	36.3% positive	<0.001
Ex-smoking	32.0% positive	34.8% positive	57.1% positive	<0.001
Wheezing in the past 12 months	40.5% positive	63.2% positive	56.3% positive	<0.001
Frequent cough	34.5% positive	43.1% positive	36.7% positive	0.040
Frequent phlegm	25.9% positive	31.5% positive	43.0% positive	<0.001
Dyspnea upon strong exertion	50.9% positive	67.9% positive	89.3% positive	<0.001
Dyspnea upon weak exertion	18.6% positive	20.6% positive	49.0% positive	<0.001

The table shows absolute numbers or percentages in case of frequencies and median values and quartiles in case of continuous parameters. The categorical variables were compared between the diagnostic groups using the chi-square statistics, while continuous parameters were compared using Kruskal–Wallis test. For the explanation of parameters, see ref. ^1^. Log_10_(s3/s2) is the logarithm of the ratio s3/s2, log_10_(s3) the logarithm of slope of phase 3. Before taking the logarithm, the values of 0.05 and 0.03, respectively, were added to account for zero values and achieve a distribution being as close to normal as possible.

### Logistic regression analyses

**Fig. 1:**
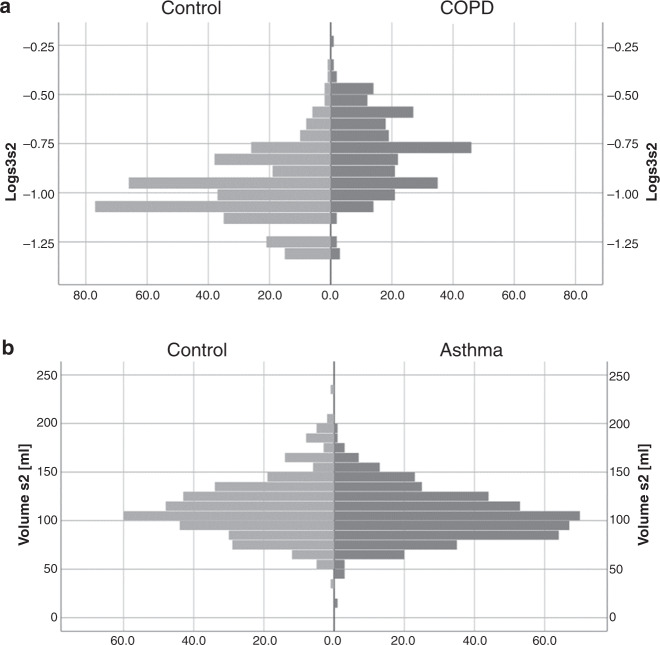
Frequency distributions of capnovolumetric parameters. Frequency distributions of the ratio s3/s2 for COPD vs control, shown as logarithm to base 10 (**a**) and of the volume of phase 2 for asthma vs control (**b**). Before taking the logarithm of the ratio s3/s2, the value of 0.05 was added to account for zero values and achieve a distribution as closely to normal as possible.

The comparison of COPD vs controls revealed dyspnea upon strong exertion, current smoking, a history of previous smoking, phlegm, the ratio of slopes of phases 3 and 2 (s3/s2), the slope of phase 3 (s3), and the ratio of area to volume of phase 3 (area/volume phase 3) as significant predictors (*p* < 0.05 each). Regarding asthma vs controls, wheezing, dyspnea upon strong exertion, current smoking, and the volume of phase 2 were predictors (*p* < 0.05 each). Regarding COPD vs asthma, wheezing, dyspnea upon both strong or mild exertion, cough, current smoking, a history of previous smoking, the ratio s3/s2, the s3, and the area/volume phase 3 were predictors (*p* < 0.05 each). The results of stepwise logistic regression analyses in terms of statistically significant odds ratios (ORs) are summarized in Supplementary Table S1. Histograms of the ratio s3/s2 for COPD vs control and of volume of phase 2 for asthma vs control are shown in Fig. 1, illustrating the significant, though small differences between the respective groups.

### Network analysis

**Fig. 2:**
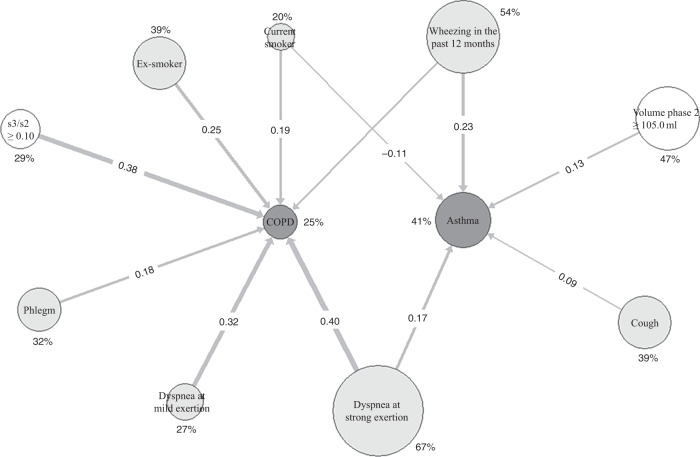
Multiple relationships of capnovolumetric parameters and clinical signs and symptoms. Quantitative network diagram comprising two capnovolumetric parameters and patients’ clinical history and symptoms. The area of the circles indicates the frequency of positive answers or positive conditions of capnovolumetric parameters compared to the cut-off values (see text). The thickness of the arrows is proportional to the respective phi-coefficients as measures of the strength of association, ranging in absolute values from 0.09 (thin line) to 0.40 (thick line) if significantly different from zero. The numerical values of phi-coefficients are given in Supplementary Table S2; the frequency of positive answers to anamnestic questions or positive capnovolumetric conditions in Supplementary Table S3. Both are indicated in the diagram.

When constructing the network diagram (Fig. 2), we used a predefined cut-off value of 0.10^1,4^ for s3/s2, and for the volume of phase 2 a cut-off value identified as optimal by receiver operator curve (ROC) analysis in the detection of asthma. The ratio s3/s2 was strongly linked to COPD and the volume of phase 2, although much weaker, to asthma. As expected, breathlessness at strong exertion was related to COPD and wheezing to asthma. Cough was related to asthma, phlegm to COPD, and smoking to both, but with opposite signs. The group of control patients was implicit in this analysis, as it served as the reference for the computation of phi-coefficients. The numerical values of the phi-coefficients are depicted in Supplementary Table S2; the frequencies of positive answers to anamnestic questions are shown in Supplementary Table S3.

### Random forest decision trees

The area under the curve (AUC) was 0.623 for the comparison of asthma vs control, corresponding to a sensitivity of 68.1% (95% confidence interval (CI) 63.5, 72.5%) and specificity of 50.3% (45.0, 55.5%). Wheezing, the volume of phase 2, dyspnea upon strong exertion, and current smoking were identified as the four most important variables. For COPD vs control, the AUC was 0.875, with sensitivity of 75.0 (69.3, 80.1%) and specificity of 83.0% (78.7, 86.7%). Area/volume of phase 3, s3/s2, dyspnea upon strong exertion, and current smoking were the four most important variables. For COPD vs asthma, the AUC was 0.880, with sensitivity and specificity of 71.2% (65.2, 76.6%) and 89.4% (86.1, 92.1%), respectively. Current smoking, s3/s2, area/volume of phase 3, and smoking history were the four most important variables.

The initial choices of the numbers of trees and variables within each tree (approximate square root of the total number of variables) were based on the default settings of the R procedure. In the next step, the parameters were tuned to establish the robustness of results. The prediction accuracy showed a plateau for a number of trees of about ≥300, thus the number of trees chosen was sufficient. Moreover, the search for the optimal number of variables used for each tree node showed that the best accuracy was obtained for three variables. Based on this, the three ensembles of trees and the sets of variables selected as important can be considered as optimal.

### Single classification and decision trees

Three single decision trees were constructed as an addition to the three ensembles, with the aim to illustrate the role of variables in single trees. To avoid small sample sizes and instability, the trees were limited to at most three generations of branches. All questions and all capnovolumetric parameters were offered to the CHAID search algorithm.

**Fig. 3:**
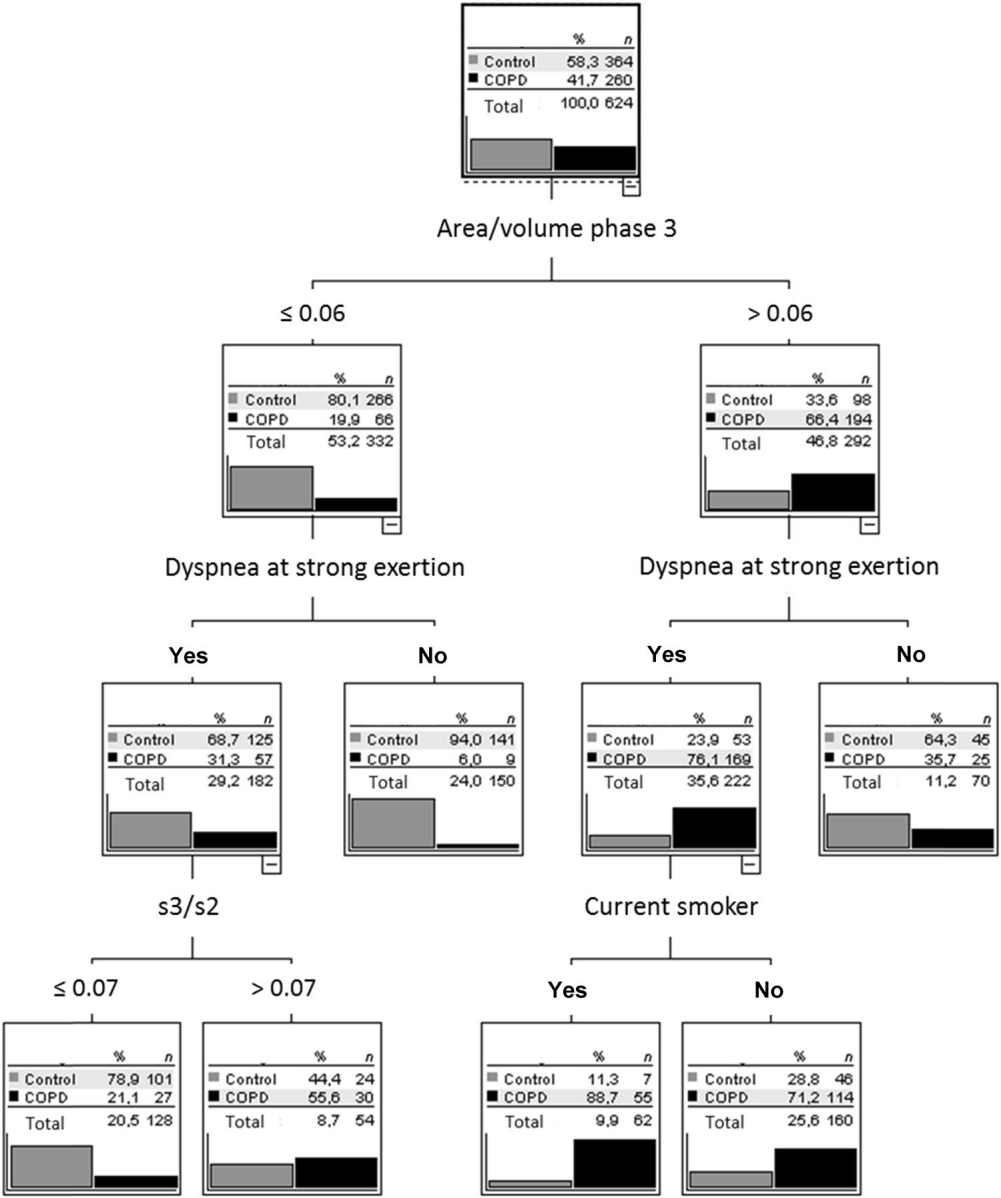
Decision tree for the comparison of COPD with control. Only patients with COPD and the control group were included. Anamnestic questions (wheezing in the past 12 months, dyspnea at strong or mild exertion, cough, phlegm, current smoker, ex-smoker) and capnovolumetric parameters (s3/s2, volume phase 2, area/volume phase 3, slope of phase 3) were offered to the algorithm (CHAID), which selected the optimal criteria. The figure shows the average result of a tenfold cross-validation.

In the decision tree for COPD vs control (Fig. 3), the four variables identified as relevant were the same as those identified in the random forest as most important. The first criterion was area/volume phase 3. If this was low, dyspnea upon strong exertion became relevant. If this was absent, COPD became very unlikely. If it was present, the ratio s3/s2 became important, whereby patients with a smaller ratio had less likely COPD. If the area/volume phase 3 was high, again dyspnea upon strong exertion was relevant. If this was present, the prevalence of COPD markedly increased, while on the next level a further increase occurred if the patient was a current smoker. Under these conditions, the prevalence of COPD increased from a baseline value of 41.7% to a final value of 88.7%. Conversely, it was as low as 6% in patients showing a low area/volume phase 3 value in the absence of dyspnea upon strong exertion. Overall, the decision tree allowed a correct classification of 77.9% of patients, with sensitivity of 76.5% (95% CI 70.9%, 81.6%) and specificity of 78.8% (74.3, 82.9%).

**Fig. 4:**
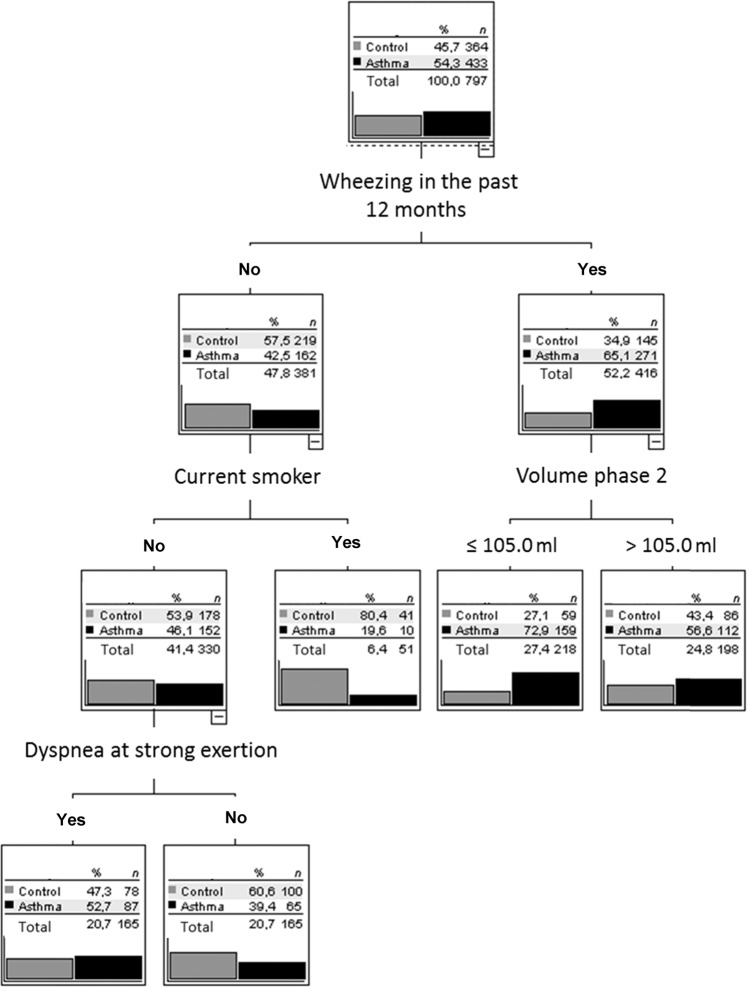
Decision tree for the comparison of asthma with control. Only patients with asthma and the control group were included. Anamnestic questions (wheezing in the past 12 months, dyspnea at strong or mild exertion, cough, phlegm, current smoker, ex-smoker) and capnovolumetric parameters (s3/s2, volume phase 2, area/volume phase 3, slope of phase 3) were offered to the algorithm (CHAID), which selected the optimal criteria. The figure shows the average result of a tenfold cross-validation.

For asthma vs control, the decision tree is shown in Fig. 4. Again, the four variables identified as important were those identified in the random forest as most important. Wheezing in the past 12 months turned out to be the dominant criterion. If answered positive, asthma was probable and the volume of phase 2 was selected as second criterion, leading to a further, though small, increase in the prevalence of asthma from a baseline value of 54.3% to a final value of 72.9%. If no wheezing was reported, the next important question was that of smoking status. If the patient was a smoker, asthma was much less likely. If the patient was a non-smoker, dyspnea at strong exertion was next informative, rendering the absence of asthma more likely in the absence of dyspnea. Overall, 62.6% of patients were correctly classified, with sensitivity of 82.6% (95% CI 78.8%, 86.1%) and specificity of 38.7% (33.7, 44.0%).

**Fig. 5:**
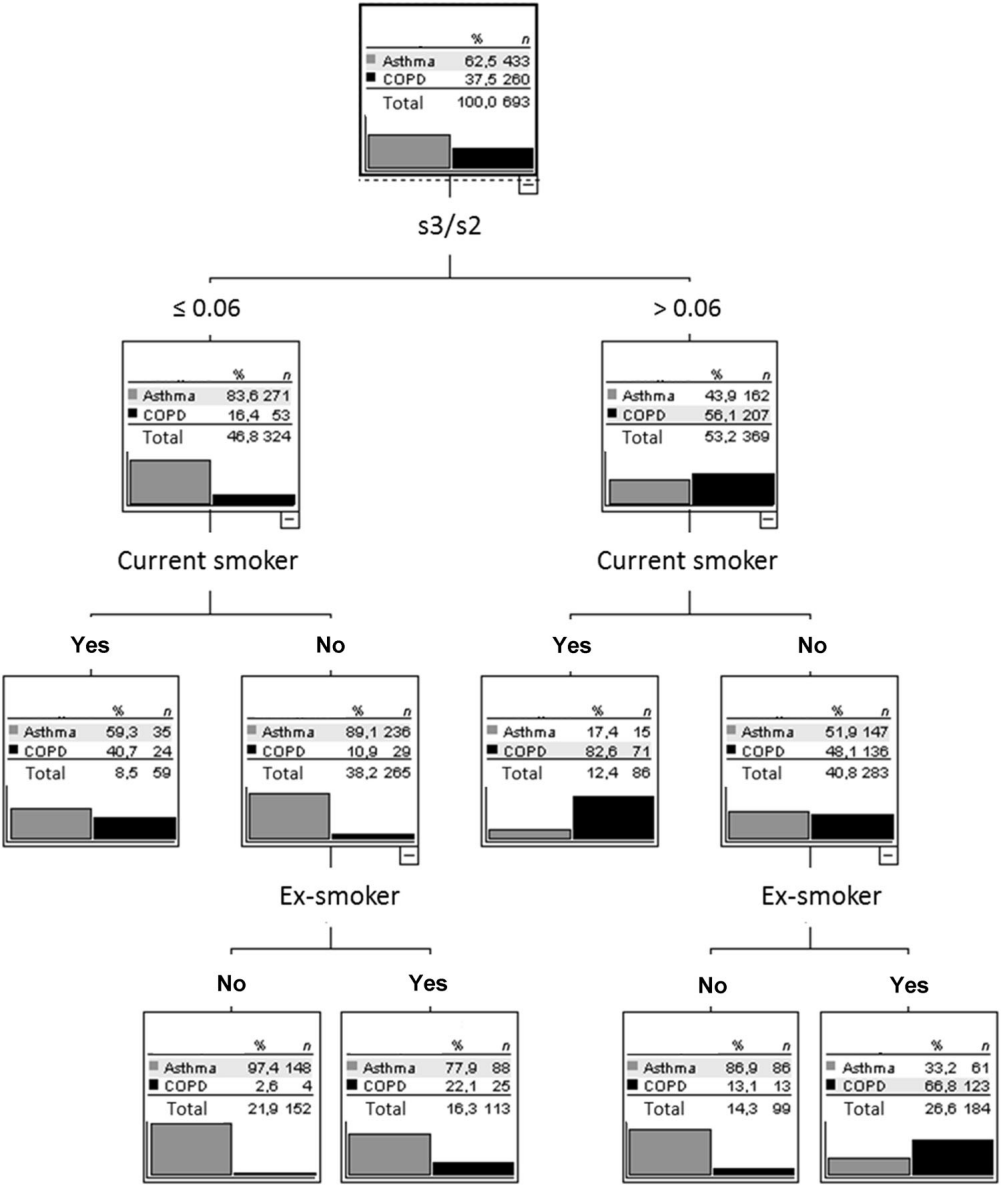
Decision tree for the comparison of asthma with COPD. Only patients with asthma or COPD were included. Anamnestic questions (wheezing in the past 12 months, dyspnea at strong or mild exertion, cough, phlegm, current smoker, ex-smoker) and capnovolumetric parameters (s3/s2, volume phase 2, area/volume phase 3, slope of phase 3) were offered to the algorithm (CHAID), which selected the optimal criteria. The figure shows the average result of a tenfold cross-validation.

Regarding the comparison between asthma and COPD, the decision tree is shown in Fig. 5. The three variables involved in the decision tree were among the four most important variables of the random forest. The first important variable was the ratio s3/s2. If this was low, the prevalence of asthma increased. It further increased on the next two levels if the patient was a never smoker. Under these conditions, the prevalence of COPD dropped from 37.5 to 2.6%. Conversely, if the ratio s3/s2 was high, the prevalence of COPD increased to 82.6%, if the patient was a current smoker. If the patient did not smoke, being an ex-smoker was associated with a higher prevalence of COPD and never smoking with a high likelihood for asthma. The results demonstrated that beyond s3/s2 the smoking status was important for further differentiation. Overall, 79.5% of patients were correctly classified, with sensitivity of 74.6% (95% CI 68.9%, 79.8%) and specificity of 82.4% (78.5, 85.9%).

Taken together, in the comparisons of COPD vs control, asthma vs control, and asthma vs COPD the random forest approach categorized 79.6, 60.0, and 82.5%, respectively, of subjects correctly, and the single decision tree 77.9, 62.6, and 79.5%, respectively.

## Discussion

The present analysis aimed at the integration of capnovolumetric parameters with symptoms and clinical history in the diagnosis of asthma and COPD. The parameters were those identified previously as relevant for the recognition of airway obstruction^1^. In a network analysis, we found the ratio s3/s2 to be related to COPD, and the volume of phase 2 to asthma, consistent with the results of logistic regression analyses and previous findings^1^. Dyspnea upon exertion, wheezing, smoking status, and phlegm were linked to COPD, while in asthma wheezing and the absence of smoking were more important, matching the expectations from clinical experience. We therefore felt justified to use our data for the development of decision trees, using the random forest approach as one of the machine learning methods, which has already been used in clinical studies^11–13^. The random forest approach was supplemented by the construction of single decision trees in order to illustrate their structure by specific examples. The results of both approaches were in very good agreement. While the single trees were more amenable to interpretation, the random forest was statistically slightly superior as judged from positive predictive values (PPVs). All trees were consistent with the network diagram and the results of our previous analysis^1^, underlining the role of the capnovolumetric parameters area/volume phase 3, ratio of slopes of phases 3 and 2 (s3/s2), and volume of phase 2; the latter is similar to the Fowler deadspace^1,14^ and inversely related to the slope of phase 2.

In the comparison of COPD with controls, the most important variable was area/volume phase 3. Given this, dyspnea upon strong exertion, the ratio s3/s2, and current smoking were relevant for the exclusion or inclusion of COPD, which seems plausible. To account for the fact that a different set of parameters was relevant, the comparison of asthma vs controls was performed separately. Noteworthy enough, the volume of phase 2 became important only when wheezing was confirmed, a low volume favoring the diagnosis of asthma, whereas in the absence of wheezing current smoking markedly decreased the likelihood of asthma. Accordingly, in the comparison of COPD with asthma current smoking, smoking history, the ratio s3/s2, and the area/volume phase 3 were most important. In the single tree, the finding that the ratio s3/s2 was the primary parameter reflected the fact that asthma patients had a low degree of airway obstruction and were similar to controls. All subsequent decisions regarding the comparison asthma vs COPD involved the current and previous smoking status, suggesting that the ratio s3/s2 comprised most of the information regarding the differential diagnosis between asthma and COPD. The cut-off values of s3/s2 within the trees were those identified as optimal for the diagnostic decisions regarding COPD, whereas the previously used^1^ value of 0.10 was pre-determined^4^ aiming at the detection of airway obstruction.

The three single trees were also valuable in demonstrating that some combinations of values were associated with marked changes in disease probability and others not. Regarding asthma vs control, for example, the absence of wheezing plus current smoking, or its presence plus low volume of phase 2, resulted in large changes. Conversely, the combination of non-smoking with dyspnea upon strong exertion, or of wheezing with a high volume of phase 2, did not markedly change the probability for asthma.

Taken together, we found that all decision trees involved at least one capnovolumetric parameter, suggesting that capnovolumetry bears relevant information in addition to clinical signs and symptoms in the clinical diagnosis of asthma and COPD. Noteworthy enough, the maximal probability to suffer from COPD as illustrated in the decision tree of Fig. 3 was similar to the PPV of spirometry to detect COPD in a general practice population^5^, while the maximal probability of asthma (Fig. 4) was equal to the PPV of bronchial provocation^15,16^. Although capnovolumetry is no substitute for spirometry, as the latter method allows to describe the severity of airway obstruction according to established criteria, our results underline its potential if no valid spirometry is available. Future studies may also combine capnovolumetry with other easily available diagnostic information to establish decision algorithms optimally combining efficiency with simplicity. According to our observation, that different sets of parameters were best for different suspected diagnoses, this probably requires separate decision algorithms, supporting the view that medical expert knowledge in terms of prior diagnostic suspicions remains indispensable.

Regarding the limitations of the study, it has to be mentioned that the present study was a secondary analysis based on previous results^1^. It included information not previously used and followed a different methodological path focusing on decision algorithms. Decision trees offer high flexibility, as different criteria can apply at each node, a complexity that in conventional regression analyses can be realized only via difficult-to-understand higher-order interaction terms. Trees suffer from overfitting, thus we used the well-known random forest ensemble approach to achieve robust and reliable results. This approach has, however, the disadvantage that the final algorithm cannot be easily depicted. To visualize the major results in a comprehensible manner, we went back to single decision trees, which were, naturally, inferior to the ensemble approach. It also should be kept in mind that especially the patients with asthma who were included in the study had been previously diagnosed using the full repertoire of diagnostic methods including bronchodilator and bronchoprovocation testing. Therefore, the diagnosis could be considered as reliable, while of course a categorization solely based on capnovolumetry cannot be more than a diagnostic hint that must be evaluated by further procedures including the response to therapy.

For an implementation of the random forest approach into clinical practice, further studies in a variety of study populations would be needed. It also would be helpful to supplement our findings by inclusion of other biomarkers, such as exhaled nitric oxide, that can be easily obtained even in primary care conditions. Unfortunately, only few ultrasound capnovolumetric devices are currently commercially available. If the method should be used in clinical practice on a broader scale, technical comparisons will also be needed. The diagnostic decision-making process might be another limitation. Thirty-seven patients received the COPD diagnosis despite showing no signs of airway obstruction. The diagnoses relied on a comprehensive assessment of the patients’ files and lung function data and had been established by a pneumologist previous to the study visit in nearly all cases. Some pneumologists might retain to the old classification COPD 0 (as a risk factor). Beyond that, in clinical practice it might occur that patients suffering from a mild form of COPD, with typical signs and symptoms, from time to time show no airway obstruction in lung function tests, using, for example, the established cut-off value of 0.7 for forced expiratory volume in 1 s/forced vital capacity; at one visit, the value may be 0.71, at another visit 0.69. Therefore, we performed a sensitivity analysis with classification of all patients without airway obstruction as “healthy controls” and found that 78.2% of patients were still correctly categorized (compared to 77.9%).

Taken together, capnovolumetry has low demands on patients’ cooperation and may be applicable in those in whom spirometry fails. Using a large diagnostic data set, we analyzed in which way capnovolumetric parameters could be combined with basic information on clinical history, signs, and symptoms to support the diagnosis and differential diagnosis of asthma and COPD. Using the approach of either single decision trees or randomized ensembles of such trees, three capnovolumetric parameters, as well as wheezing, dyspnea upon strong exertion, and smoking history, turned out to be most relevant. Our findings underline the usefulness of capnovolumetry as an additional tool in the diagnostic assessment of asthma and COPD.

## Methods

### Patients

The analysis used data from a previous study performed in a private clinical practice in Augsburg, Germany, in which capnovolumetry was performed as index test, while the presence or absence of airway obstruction was evaluated via spirometry and bodyplethysmography as a reference standard^1^. The physician-based diagnoses relied on a comprehensive assessment of the patients’ files and lung function data. No other inclusion or exclusion criteria were used. Patients with COPD and the comorbidity asthma (*n* = 34) were assigned to the COPD group, as this disease dominated the functional alterations. The study had been approved by the Ethical Committee of the Medical Faculty of the Technical University of Munich, and all patients gave their written, informed consent. The original study is registered under DRKS00013935 at German Clinical Trials Register (DRKS) where the study protocol can be accessed.

### Assessments

Capnovolumetry was performed during tidal breathing over at least ten breathing cycles, with the only instruction to avoid deep breaths or panting, and the last five cycles were evaluated as mean values. The time course of expiratory CO_2_ was determined via ultrasound via determination of the molar mass (SpiroScout, software LFX 1.8.0, Ganshorn, Niederlauer, Germany), whereby the parameters describing the capnovolumetric curves were computed by the built-in software. The four parameters previously identified as most relevant for the detection of airway obstruction were the s3, s3/s2, the volume of phase 2, and area/volume phase 3^1^. The capnovolumetric parameters describe the form of the expiratory CO_2_ curve plotted against expiratory volume. The initial phase 1 comprises the dead space and is characterized by a CO_2_ concentration near zero. It is followed by a steep rise of CO_2_ concentration (with slope s2) in phase 2, as a result of the mixing of CO_2_-free air with alveolar gas within the bronchial volume. Phase 2 is followed by phase 3 that represents the alveolar compartment and shows a slope of CO_2_ concentration (s3) that is less than the slope of phase 2. In the presence of emphysema, slope 3 increases and slope 2 decreases, both primarily due to inhomogeneity of ventilation, thereby leading to a marked increase in the ratio s3/s2. In asthma, there is at least a tendency for a reduction of slope 2 and the volume of phase 2, both of which are indicative of (residual) bronchial obstruction. Area/volume phase 3 is closely related to alveolar ventilation, especially alveolar dead space, and therefore valuable particularly for the recognition of COPD.

For the comprehensive assessment of clinical history, signs, and symptoms, a questionnaire covering seven questions regarding dyspnea upon mild or strong exertion, cough, phlegm, wheezing, and smoking status (current, ex-smoker) was used (see Supplementary Methods for the questions).

### Statistical analysis

As we aimed at the evaluation of capnovolumetry, only functional data from this measurement were used. Median values and quartiles were computed for patients’ description, and binary logistic regression analyses were performed for the comparison of the COPD with the control group, asthma with control, and COPD with asthma. We relied on these binary distinctions, as the sets of relevant parameters turned out to be different and ternary comparisons resulted in complicated and non-robust predictive models.

In a next step, a quantitative network diagram describing the multiple relationships between parameters was constructed, using an adjacency matrix based on the strength of associations (phi-coefficients; control group as reference). This diagram comprised the anamnestic information as binary variables; moreover, binary categorizations of the ratio of slopes s3/s2 and the volume of phase 2 could be correlated with the binary results of the questions. These two parameters were chosen among the parameters of capnovolumetry in order to limit the complexity of the diagram. In the diagram, the area of the circles indicates the frequency of positive answers or capnovolumetric conditions, respectively, and the thickness of the arrows indicates the strength of association (phi-coefficient). For construction, the statistical software R was used^17^.

While illustrating different associations for asthma and COPD, the network did not provide a decision algorithm. This was achieved by systematic generation of ensembles of binary classification and decision trees using the random forest approach and taking the majority vote of trees as outcome. Separate ensembles were constructed for COPD vs control, asthma vs control, and COPD vs asthma. Consistent with the fact that the sets of relevant predictors for these three comparisons were different (see above), comparisons comprising all three groups resulted in non-robust, difficult-to-interpret results and were thus not further evaluated. Following established procedures^18^, the trees were constructed from the data by random selection of patients’ subsets (*n* = 500) and sets of variables at each node (mtry = 3). All seven questions and all four continuous capnovolumetric parameters (without pre-defined cut-off values) were offered to the search algorithm. The patients not included in a specific tree (out of bag) allowed the evaluation of accuracy in terms of 2 × 2 confusion matrices and ROCs, yielding sensitivity, specificity, and AUC. The relative importance of parameters was described by the computed mean decrease in accuracy as well as the GINI criterion^18^.

While random forests have the advantage of reducing problems arising from overfitting, they have the disadvantage that the ensembles of trees can be described only statistically. Therefore, a parallel construction of single trees by a classical procedure might be helpful for illustration and interpretation, in particular if the single trees comprise most or all of the variables identified as important in the ensembles. For this purpose, we used the CHAID method as implemented in SPSS^10^, including Bonferroni correction and tenfold cross-validation. Again, separate trees were constructed for COPD vs control, asthma vs control, and COPD vs asthma.

A more detailed description of the statistical methods can be found in Supplementary Methods. All statistical analyses were performed with SPSS (Version 25, IBM Corp., Armonk, NY, USA) and the module “randomForest” from the software package R^19^. The level of significance was assumed at *p* < 0.05.

### Reporting summary

Further information on research design is available in the Nature Research Reporting Summary linked to this article.

## Supplementary information

Supplementary Information

Reporting Summary

## Data Availability

The data set analyzed during the current study is available from the corresponding author on reasonable request.
