# Supplementary material for: Capnovolumetry in combination with clinical history for the diagnosis of asthma and COPD
Source: NPJ Prim Care Respir Med. 2020 Jul 30;30:32. doi: 10.1038/s41533-020-00190-z (PMC7393160; doi:10.1038/s41533-020-00190-z)
Supplement: Supplementary file 1 — Supplementary Information [file 41533_2020_190_MOESM1_ESM.pdf]

SUPPLEMENT

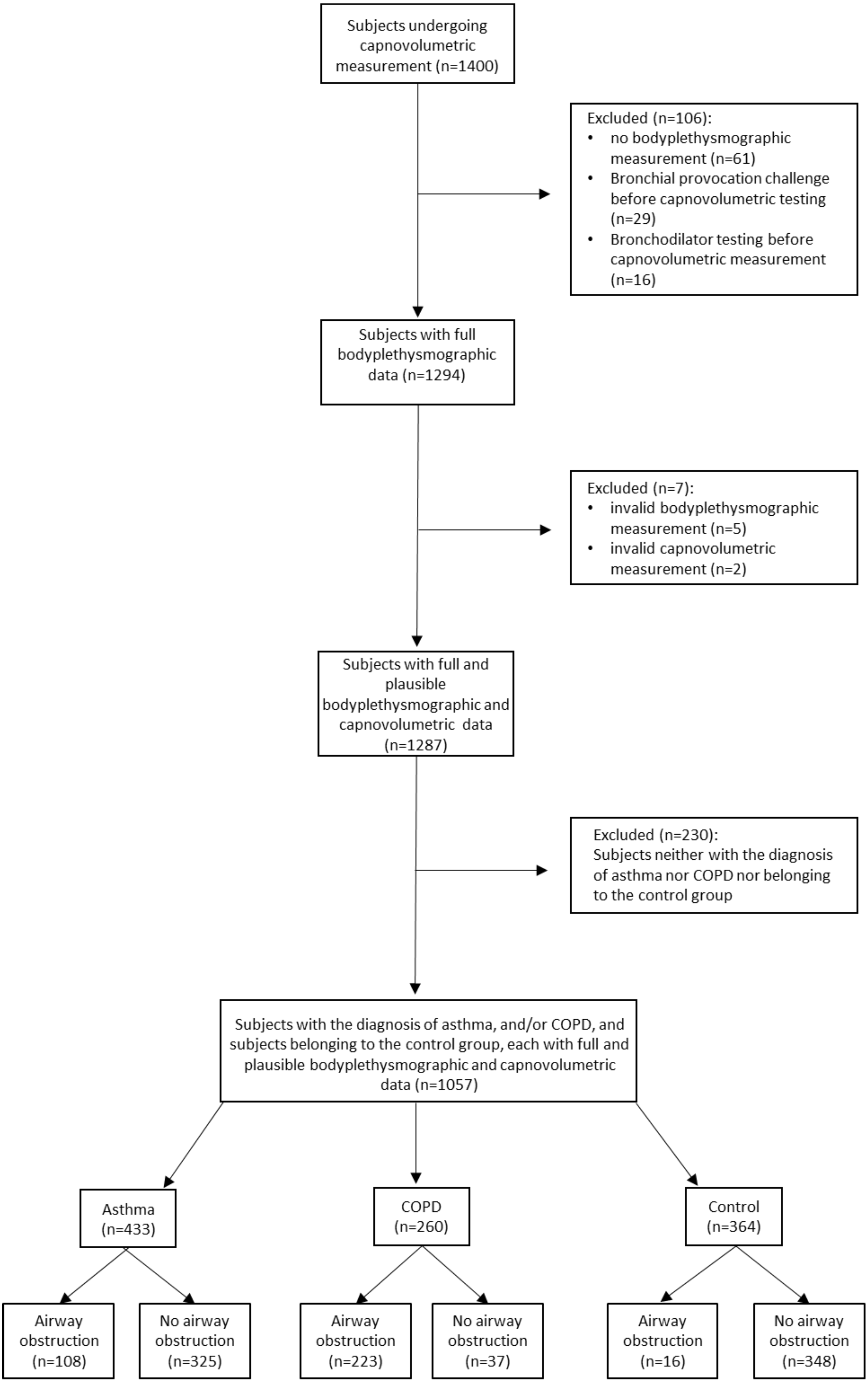

Subjects undergoing capnovolumetric measurement (n=1400)
Excluded (n=106):
• no bodyplethysmographic measurement (n=61)
• Bronchial provocation challenge before capnovolumetric testing (n=29)
• Bronchodilator testing before capnovolumetric measurement (n=16)
Subjects with full bodyplethysmographic data (n=1294)
Excluded (n=7):
• invalid bodyplethysmographic measurement (n=5)
• invalid capnovolumetric measurement (n=2)
Subjects with full and plausible bodyplethysmographic and capnovolumetric data (n=1287)
Excluded (n=230):
Subjects neither with the diagnosis of asthma nor COPD nor belonging to the control group
Subjects with the diagnosis of asthma, and/or COPD, and subjects belonging to the control group, each with full and plausible bodyplethysmographic and capnovolumetric data (n=1057)
Asthma (n=433)
COPD (n=260)
Control (n=364)
Airway obstruction (n=108)
No airway obstruction (n=325)
Airway obstruction (n=223)
No airway obstruction (n=37)
Airway obstruction (n=16)
No airway obstruction (n=348)

Supplementary Figure S1. Flow-chart of the selection process leading to a subset of 1057 patients included into the present analysis. A total of 1400 consecutive patients underwent capnovolumetry. Patients who turned out to have had bronchial provocation challenges or bronchodilator testing prior to capnovolumetry due to organizational reasons were excluded (n=45). Moreover, patients who did not undergo bodyplethysmographic and spirometric measurements (n=61) were excluded. Five patients were excluded due to low quality of their bodyplethysmographic measurement data, and two patients based on invalid capnovolumetric measurements. For the present analysis patients were selected who had a diagnosis of COPD or asthma (potentially both), or did not show any respiratory disease (control subjects). Those with the diagnosis of other respiratory diseases (such as restrictive disorders, pneumonia or other infections, pleural diseases, lung tumor, bronchiectasis) were excluded (n=230), leading to a final subset of 1057 patients.

Supplementary table S1

The table shows the results of stepwise logistic regression analyses in terms of the statistically significant odds ratios (OR). The OR refer to the first versus second condition for the three comparisons of diagnoses shown. In case of answers to questions, a positive answer is associated with the OR given. In case of continuous variables from capnovolumetry, the OR for an interquartile change in the study population (COPD, asthma, control) is shown to ensure the comparability within the table. Values that were not statistically significant are indicated by a hyphen.

	**COPD vs Control**	**Asthma vs Control**	**COPD vs Asthma**
Phase 3 (s3)	0.566	-	1.618
s3/s2	2.285	-	2.151
Area/volume phase 3	3.212	-	2.507
Volume phase 2	-	0.775	-
Current smoker	12.773	0.488	30.857
Ex-smoker	7.988	-	7.868
Frequent cough	-	-	0.605
Frequent phlegm	1.748	-	-
Wheezing in the last 12 months	-	2.378	0.426
Dyspnea at strong exertion	7.037	1.440	3.063
Dyspnea at mild exertion	-	-	2.696

Supplementary Table S2

Phi-coefficients as measures of association for two binary variables. Binary results (yes/no) of anamnestic questions or capnovolumetric conditions were analyzed for their relationship to asthma or COPD, taking the control group as reference. For the ratio of s3/s2 a predefined cut-off value (0.10) was used. The cut-off value of 105.0 ml for the volume of phase 2 was identified by ROC analysis of asthma vs control as optimal, using this parameter as a single predictor. Only significant phi-coefficients are listed. The phi-coefficients were used for the construction of a quantitative network diagram (figure 2). n.s. = not significant.

**Asthma - Control**	
**Questions (binary results (yes/no))**	**Phi-coefficient**
Wheezing in the last 12 months?	0.23
Dyspnea at mild exertion?	n.s.
Dyspnea at strong exertion?	0.17
Current smoker?	-0.11
Ex-smoker?	n.s.
Frequently coughing?	0.09
Increased phlegm?	n.s.
Ratio s3/s2 ≥ 0.10	n.s.
Volume of phase 2 ≤ 105.0ml	0.13
**COPD - Control**	
**Questions (binary results (yes/no))**	**Phi-coefficient**
Wheezing in the last 12 months?	0.16
Dyspnea at mild exertion?	0.32
Dyspnea at strong exertion?	0.40
Current smoker?	0.19
Ex-smoker?	0.25
Frequently coughing?	n.s.
Increased phlegm?	0.18
Ratio s3/s2 ≥ 0.10	0.38
Volume of phase 2 ≤ 105.0ml	n.s.

Supplementary Table S3.

Frequency of positive answers to anamnestic questions or capnovolumetric conditions. In addition, the prevalence of the diagnoses of asthma and COPD in the study population is indicated. For the ratio of s3/s2 a predefined cut-off value (0.10) was used. The cut-off value of 105.0 ml for the volume of phase 2 was identified by ROC analysis (see legend table S1). The values were used in the quantitative network diagram shown in figure 2.

**Anamnestic questions/ capnovolumetric conditions/ diagnoses**	**Frequency of positive answers (%)**
Wheezing in the last 12 months?	53.7
Dyspnea at mild exertion?	26.9
Dyspnea at strong exertion?	67.4
Current smoker?	20.3
Ex smoker?	39.4
Frequently coughing?	38.6
Increased phlegm?	32.4
Ratio s3/s2 $\geq$ 0.10	28.9
Volume of phase 2 $\leq$ 105.0ml	47.4
Asthma	41.0
COPD	24.6

Supplementary Methods

Short description of the statistical methods

Logistic regression analysis

This is a modification of the well-known linear regression analysis in the sense that the dependence of an outcome variable of a number or predictor variables is determined. The predictor variables can be continuous or categorical, especially binary. The difference to linear regression analysis is that the outcome variable is categorical, usually binary. A typical binary comparison is that between two diagnoses, a typical binary predictor “smoking Yes/No”, and a typical continuous predictor a lung function measure. The dependence on the predictors is determined simultaneously, i.e. not separately for each of them. The degree of dependence can be expressed through a coefficient in analogy to linear regression. However, as this occurs in an exponential function, it is commonly expressed as odds ratio giving the relative chance of one of the outcomes relative to the other for a change in one unit of the predictors. The latter may be a Yes/No change in case of a binary predictor. The practical use is to multiply the respective odds ratios if their conditions are met and thus to derive an estimate for the likelihood of one diagnosis over the alternative, provided the model is correct.

Network analysis

This is a way to depict relationships and their strength among a set of binary variables, such as Yes/No answers. Continuous variables are dichotomized using a pre-defined cut-off value and can thereby be included. Essentially, the relationship between variables is determined in 2x2 contingency tables and expressed as phi-coefficient that can be interpreted as correlation coefficient. The frequency of each condition is commonly indicated by the size of circles representing the variables, and the strength of each relationship by the thickness of the respective line. This approach provides a convenient visualization of the overall findings, however without a deeper analysis in terms of multiple simultaneous relationships or the prediction of diagnoses.

Decision trees

We assume that we aim at a decision between two diagnoses based on a set of variables. The first step is to identify the most relevant variable for decision, i.e. the variable yielding the best separation between individuals regarding their categorization into two groups. The criterion for this is either fixed, e.g. Yes/No, or, for continuous variables, identified via the optimal cut-off value. This results in a categorization of individuals into two groups, one comprising those in which the criterion is met, the other those in whom it is not met. Each of the two groups is then analyzed in exactly the same way, the resulting groups again, and so on, until an end criterion is met, e.g. the size of the groups is smaller than some value. Thus, a decision is made at each node, which results in a branching. In our approach, we allowed only for two branches to keep the model simple. It is important to note that in each of parallel branches different variables may be relevant, a fact which allows for a great flexibility of decision trees. The commonly used algorithms differ by technical details, for example in the way, which variables are selected, whether variables can be used repeatedly throughout a branch or are “used up” if they occurred once in a branch. The CHAID algorithm used by us is based on a chi-square statistics computed at each node and does not use a variable again in a branch if it had already been used. This reduces the risk of overfitting in the sense that trees tend to fit the data from which they are constructed very well but are less reliable when applied to new data. The advantage of the decision tree is that it can be used directly by inserting a patient’s values at each node and looking for the final result of the decision sequence.

Random forest

Decision trees are easy to visualize, understand and use, but known to be susceptible to errors resulting from overfitting. To increase the robustness, an ensemble of trees can be constructed that work in parallel, using their majority vote for decision. The construction of each tree can be based on a subset of the data which is sampled by a random procedure from the total data set, typically with

the possibility to draw each individual's data set repeatedly. Moreover, at each branch, a random subset of variables is offered to the algorithm searching for the optimal variable (and possibly optimal cut-off value for continuous variables); the algorithms typically work as described above for decision trees. This approach has been shown to be remarkably statistically robust and safe against overfitting, while at the same time in many cases yielding better results than a single tree. The disadvantage is that the ensemble of trees that are constructed (typically of the size 500-1000) cannot be visualized, naturally, and also cannot be listed in a compact form that is directly useful for a reader. It can only be realized as software algorithm, and the characteristics of the trees can only be described statistically in terms of the distributions of variables and nodes. This was the reason why we additionally identified a single optimal tree. The fact that this tree contained just the variables that had been identified as most important in the random forest approach and that the reliability in the recognition of diagnoses was only slightly less, underlined the consistency of our data set and analyses.
