# Supplementary material for: Capnovolumetry in combination with clinical history for the diagnosis of asthma and COPD
Source: NPJ Prim Care Respir Med. 2020 Jul 30;30:32. doi: 10.1038/s41533-020-00190-z (PMC7393160; doi:10.1038/s41533-020-00190-z)
Supplement: Supplementary file 2 — Reporting Summary [file 41533_2020_190_MOESM2_ESM.pdf]

nature research

Corresponding author(s): DBPR NPJPCRM-00889

Last updated by author(s): Jun 17, 2020

Reporting Summary

Nature Research wishes to improve the reproducibility of the work that we publish. This form provides structure for consistency and transparency in reporting. For further information on Nature Research policies, see our Editorial Policies and the Editorial Policy Checklist.

Statistics

For all statistical analyses, confirm that the following items are present in the figure legend, table legend, main text, or Methods section.

n/a	Confirmed	
☐	☒	The exact sample size (*n*) for each experimental group/condition, given as a discrete number and unit of measurement
☒	☐	A statement on whether measurements were taken from distinct samples or whether the same sample was measured repeatedly
☐	☒	The statistical test(s) used AND whether they are one- or two-sided<br>*Only common tests should be described solely by name; describe more complex techniques in the Methods section.*
☐	☒	A description of all covariates tested
☒	☐	A description of any assumptions or corrections, such as tests of normality and adjustment for multiple comparisons
☐	☒	A full description of the statistical parameters including central tendency (e.g. means) or other basic estimates (e.g. regression coefficient) AND variation (e.g. standard deviation) or associated estimates of uncertainty (e.g. confidence intervals)
☐	☒	For null hypothesis testing, the test statistic (e.g. *F*, *t*, *r*) with confidence intervals, effect sizes, degrees of freedom and *P* value noted<br>*Give P values as exact values whenever suitable.*
☒	☐	For Bayesian analysis, information on the choice of priors and Markov chain Monte Carlo settings
☒	☐	For hierarchical and complex designs, identification of the appropriate level for tests and full reporting of outcomes
☐	☒	Estimates of effect sizes (e.g. Cohen's *d*, Pearson's *r*), indicating how they were calculated

Our web collection on statistics for biologists contains articles on many of the points above.

Software and code

Policy information about availability of computer code

Data collection	We did not use software for data collection.
Data analysis	We did not use unreported custom computer code or algorithm.

For manuscripts utilizing custom algorithms or software that are central to the research but not yet described in published literature, software must be made available to editors and reviewers. We strongly encourage code deposition in a community repository (e.g. GitHub). See the Nature Research guidelines for submitting code & software for further information.

Data

Policy information about availability of data

All manuscripts must include a data availability statement. This statement should provide the following information, where applicable:

- Accession codes, unique identifiers, or web links for publicly available datasets
- A list of figures that have associated raw data
- A description of any restrictions on data availability

The dataset analysed during the current study is available from the corresponding author on reasonable request.

Field-specific reporting

Life sciences study design

All studies must disclose on these points even when the disclosure is negative.

Sample size	The analysis used data from a previous study performed in a private clinical practice in Augsburg, Germany, in which capnovolumetry was performed as index test, while the presence or absence of airway obstruction was evaluated via spirometry and bodyplethysmography as a reference standard. Based on previous studies, we expected a prevalence of airway obstruction of 20%. In a pilot study using the ratio of slopes of phases 3 and 2 (s3/s2), the sensitivity was 90% and the specificity 86% for the detection of airway obstruction at the cut-off s3/s2≥ 0.10. A power calculation based on these assumptions revealed that at least 1280 patients were needed to establish a sensitivity and specificity of 80% each with a 95% confidence interval of ±5%. We expected incomplete data in about 10% of patients and therefore aimed to include 1400 patients.
Data exclusions	A total of 1400 consecutive patients underwent capnovolumetry. Patients who turned out to have had bronchial provocation challenges or bronchodilator testing prior to capnovolumetry due to organizational reasons were excluded (n=45). Moreover, patients who did not undergo bodyplethysmographic and spirometric measurements (n=61) were excluded. Five patients were excluded due to low quality of their bodyplethysmographic measurement data, and two patients based on invalid capnovolumetric measurements. For the present analysis patients were selected who had a diagnosis of COPD or asthma (potentially both), or did not show any respiratory disease (control subjects). Those with the diagnosis of other respiratory diseases (such as restrictive disorders, pneumonia or other infections, pleural diseases, lung tumor, bronchiectasis) were excluded (n=230), leading to a final subset of 1057 patients.
Replication	This aspect is not applicable. We analyzed data from a diagnostic study based on capnovolumetry and basic anamnestic information.
Randomization	The analysis used data from a previous diagnostic study performed in a private clinical practice in Augsburg, Germany, in which capnovolumetry was performed as index test, while the presence or absence of airway obstruction was evaluated via spirometry and bodyplethysmography as a reference standard. The study did not contain a control group and is therefore a non-randomised study.
Blinding	The pneumologists who established the diagnoses were blinded to the results of capnovolumetric measurements. The diagnoses relied on a comprehensive assessment of the patients' clincial histories and lung function data.

Reporting for specific materials, systems and methods

We require information from authors about some types of materials, experimental systems and methods used in many studies. Here, indicate whether each material, system or method listed is relevant to your study. If you are not sure if a list item applies to your research, read the appropriate section before selecting a response.

Materials & experimental systems

n/a	Involved in the study
☒	☐ Antibodies
☒	☐ Eukaryotic cell lines
☒	☐ Palaeontology and archaeology
☒	☐ Animals and other organisms
☐	☒ Human research participants
☐	☒ Clinical data
☒	☐ Dual use research of concern

Methods

n/a	Involved in the study
☒	☐ ChIP-seq
☒	☐ Flow cytometry
☒	☐ MRI-based neuroimaging

Human research participants

Policy information about studies involving human research participants

Population characteristics	Overall, 1057 patients fulfilled the inclusion criteria (see above), among them 567 females and 490 males, with mean age 56 years; 433 patients had a diagnosis of asthma, 260 a diagnosis of COPD, and 364 were control patients. Based on the lung function data, 347 patients had airway obstruction (of these 30% suffered from asthma, 66% from COPD, and 4% belonged to the control group).
Recruitment	We included 1400 consecutive patients attending a private clinical practice in Augsburg for their first diagnostic work-up or follow-up evaluations and giving oral and written informed consent. Exclusion criteria were age less than 18years and/or inability to understand the German language, without further requirements.
Ethics oversight	The study had been approved by the Ethical Committee of the Medical Faculty of the Technical University of Munich, Germany.

Note that full information on the approval of the study protocol must also be provided in the manuscript.

Clinical data

Policy information about clinical studies

All manuscripts should comply with the ICMJE guidelines for publication of clinical research and a completed CONSORT checklist must be included with all submissions.

Clinical trial registration	The original study is registered under DRKS00013935 at German Clinical Trials Register (DRKS).
Study protocol	The study is registered under DRKS00013935 at German Clinical Trials Register (DRKS) where the study protocol can be accessed.
Data collection	This diagnostic study was performed between February and April 2018 in a pulmonary outpatient clinic led by six pneumologists in Augsburg (Germany). During this period lung function data, results of capnovolumetric measurements and the diagnoses of patients documented on patient's files were collected.
Outcomes	As a primary outcome we aimed to quantify the diagnostic accuracy of capnovolumetry for the recognition of airway obstruction using the ratio of slopes in capnovolumetry as primary parameter in a large sample of unselected patients under ambulatory care conditions (see "Diagnostic accuracy of capnovolumetry for the identification of airway obstruction – results of a diagnostic study in ambulatory care", Kellerer et. al, 2019). In the present analyses, we investigated as a secondary outcome, in which way capnovolumetric parameters can be combined with basic anamnestic information to support the diagnosis of asthma and COPD.
